# Demonstration of central conduction time and neuroplastic changes after cervical lordosis rehabilitation in asymptomatic subjects: a randomized, placebo-controlled trial

**DOI:** 10.1038/s41598-021-94548-z

**Published:** 2021-07-28

**Authors:** Ibrahim M. Moustafa, Aliaa A. Diab, Fatma Hegazy, Deed E. Harrison

**Affiliations:** 1 Department of Physiotherapy, College of Health Sciences, University of Sharjah, Sharjah, UAE; 2 Basic Science Department, Faculty of Physical Therapy, Cairo University, Giza, Egypt; 3 CBP Nonprofit (A Spine Research Foundation), 950 E. Riverside Drive, Eagle, ID USA

**Keywords:** Medical research, Neurology

## Abstract

A randomized controlled study was conducted to evaluate the effect of rehabilitation of the cervical sagittal configuration on sensorimotor integration and central conduction time in an asymptomatic population. Eighty (32 female) participants with radiographic cervical hypolordosis and anterior head translation posture were randomly assigned to either a control or an experimental group. The experimental group received the Denneroll cervical traction while the control group received a placebo treatment. Interventions were applied 3 × per week for 10 weeks. Outcome measures included radiographic measured anterior head translation distance, cervical lordosis (posterior bodies of C2–C7), central somatosensory conduction time (latency) (N13–N20), and amplitudes of potentials for spinal N13, brainstem P14, parietal N20 and P27, and frontal N30. Outcomes were obtained at: baseline, after 10 weeks of intervention, and at 3 months follow up. After 10 weeks and 3-months, between-group analyses revealed statistically significant differences between the groups for the following measured variables: lordosis C2–C7, anterior head translation, amplitudes of spinal N13, brainstem P14, parietal N20 and P27, frontal N30 potentials (*P* < 0.001), and conduction time N13–N20 (*P* = 0.004). Significant correlation between the sagittal alignment and measured variables were found (*P* < 0.005). These findings indicate restoration of cervical sagittal alignment has a direct influence on the central conduction time in an asymptomatic population.

## Introduction

Disorders of the cervical spine are among the greatest contributors to spine pain, disability, and work loss worldwide^1^. These disorders are generally episodic with considerable variation in symptomatology^2^. There are also wide variations in assessment methods and treatment approaches for patients presenting with cervical spine disorders^3–5^. Problematically, most treatments for neck disorders have limited efficacy and this is particularly evident after long-term, post-therapeutic follow-up^3–5^. Although there is general agreement regarding the need of conservative treatment for cervical spine abnormalities, the precise treatment protocols for the best results and when to use them for the various disorders remains unanswered^1–5^.

Cervical spine alignment has been shown to be significantly related to patient outcomes; this has been particularly substantiated within the surgical literature^6–8^, but also in manual therapy^9–11^. Cervical kyphosis is considered a spinal deformity and is associated with pain and disability^6–8,12^. Altered cervical spine curvatures are associated with craniocervical symptoms including: headache^13,14^, migraine^14,15^, radiculopathy and myelopathy^16,17^. Similarly, the magnitude of forward head posture (FHP) is highly associated with neck pain^18^. Recently, FHP has been identified to be associated with altered cervical sensorimotor control and autonomic nervous system function^19^. Thus, in addition to pain syndromes, abnormal cervical sagittal alignment appears to play a role in altered neurophysiological changes in somato-sensory processing and sensori-motor control^19,20^.

Neuroplasticity is an inherent ability of the brain and nervous system to develop and modify synaptic connections resulting in changes in structure, function, and hierarchal organization within the nervous system. Neuroplastic changes occur across the lifespan of an organism due to repetitive and new activities, learning, and from major or minor traumatic injuries^21–23^. Pain is considered one of the strongest contributing factors triggering neuroplastic changes. Many studies suggest that these neuroplastic changes are an adaptive process due to continuous and noxious nociceptive impulses into the spinal, sub-cortical and cortical areas of the central nervous system^24–26^.

There exists an interdependent link between the reorganization of the cortex on one hand and pain on the other, indicating that these maladaptive changes appear to restore function and decrease pain perception intensity^27,28^. Of interest, altered afferent input is considered one of best explanations for the development of abnormal neuro-plastic alterations in the central nervous system (CNS)^29–32^. According to the above review, the assumption that restoring normal sagittal plane posture and cervical spine alignment would be an important consideration for a better afferentation process, however, studies addressing this relationship are speculative or do not exist to our knowledge.

Neuroplastic changes might explain relevant specific abnormalities and features identified in presenting populations with cervical sagittal malalignment^6–8,16,17^. Controlled studies, however, evaluating the effect of the cervical sagittal configuration on sensorimotor integration and central conduction time are lacking. Problematically, investigations into symptomatic participants with known dysfunction and moderate to extensive spine degenerative changes would likely confound any findings that might be specifically due to altered sagittal alignment. Accordingly, in the current study, we address three main questions in a young adult, asymptomatic and otherwise healthy population: (1) whether cervical sagittal alignment without the presence of pain might yield neuro-plastic alterations in the somatosensory system; (2) where does the possible induced modulation take place; and (3) to identify any role that sagittal cervical alignment improvement plays in somatosensory central conduction. We hypothesize that improvement in sagittal cervical alignment will positively alter somatosensory central conduction and central processing.

## Methods

A prospective, investigator-blinded, parallel-group, randomized clinical trial was performed at the clinical research facility of our university. This trial was registered with the Pan African Clinical Trial Registry (PACTR201703002068409 dated 02/03/2017). Participant recruitment began following approval from the Ethics Committee of the Faculty of Physical Therapy, Cairo University. Informed consents were provided to and obtained from all participants and/or their legal guardians prior to data collection in accordance with relevant guidelines and regulations. Participants were collected as a convenience sample of asymptomatic university attendees and friends and family members of patients attending the outpatient clinics at our university.

Both male and female participants were included in this study if they were aged between 18 to 25 years and had no current or previous complaints of neck pain within the past year. Cervical spine X-rays (lateral views) were obtained for each participant in the natural standing position. Participants were then screened prior to inclusion for magnitudes of their radiographic determined cervical lordosis (absolute rotation angle ARA C2–C7 using the angle of intersection of two lines drawn along the posterior margins of the vertebral body of C2 and C7, respectively)^33,34^. A measurement of anterior head translation (AHT) was assessed using a vertical line originating from the posterior inferior body corner of C7 and measuring the horizontal offset of the posterior superior corner of C2 relative to this vertical line. Participants were included if the AHT distance was more than 15 mm and ARA C2–C7 was less than 25° as this is one standard deviation below the mean value reported by Harrison et al^33^. The reliability of these measurements are excellent. The ARA C2–C7 measurement has an estimated inter-examiner correlation coefficient of 0.94 (range of 0.90–0.97) and intra-examiner correlation coefficient of 0.97 (range of 0.95–0.99). For AHT measurements, both inter-examiner and intra-examiner reliability correlation coefficients have been reported to be between 0.98 and 1.0. Furthermore, the standard error of measurement has been reported to be between 1° and 2° for the ARA C2–C7 and 1–2 mm for the measurement of AHT^34^.

Participants were screened for the following exclusion criteria: (i) inflammatory joint disease or other systemic pathologies; (ii) prior history of overt injury and surgery relating to the musculoskeletal system, or disorder related to the spine and extremities; (iii) musculoskeletal pain in the last 3 months. Participants were randomized to either the intervention group (40) or a control group (40). A person independent of the study assigned participant numbers from a sealed envelope method containing random numbers. Using a method of random permuted blocks of different sizes, each block was placed in a sequence of consecutively numbered sealed envelopes, which were stored in a locked location. As participants were entered into the trial, the investigator opened a new envelope in its proper sequence as witnessed by each participant.

### Denneroll extension traction

Participants in the intervention group received the Denneroll cervical orthotic (Denneroll Industries, Sydney, Australia; http://www.denneroll.com). In the current study, Denneroll cervical traction was used to restore the normal cervical alignment. This intervention was utilized because 3-ponit bending traction procedures have been found to improve cervical sagittal alignment in several previous studies^20,35–37^. We followed previously published protocols and procedures for application of this orthotic^35–37^. The Denneroll orthotic was performed in the physiotherapy clinic setting. The participants were instructed to lie supine on the floor, in a straightened position, with their arms gently folded across their stomach. The Denneroll was placed on the ground and the examiner positioned the apex of the Denneroll in one of two regions: the mid cervical spine (C4–C5) or the lower cervical spine (C6–T1) depending on the location of the apex or most kyphotic/straightened region of each participant’s cervical curvature deformity. Placement of the Denneroll at the apex of the cervical curve abnormality achieves maximum extension bending in that region. Note, both radiographic measurement and visualization of the cervical curvature abnormality on each participant’s spine radiograph was used to determine Denneroll placement^35–37^.

### Control group placebo intervention” Sham traction”

To account for the attention time difference, therapist contact, support, and expectations, the control group was treated with a placebo treatment using a folded or partially rolled, standard cervical hydrocollator towel located in the mid or lower cervical spine as an intervention to mimic the Denneroll orthotic traction. The ridges on the hydrocollator towel mimic the fulcrum bumps on the Denneroll orthotic, without applying significant extension bending of the cervical spine.

**Figure 1:**
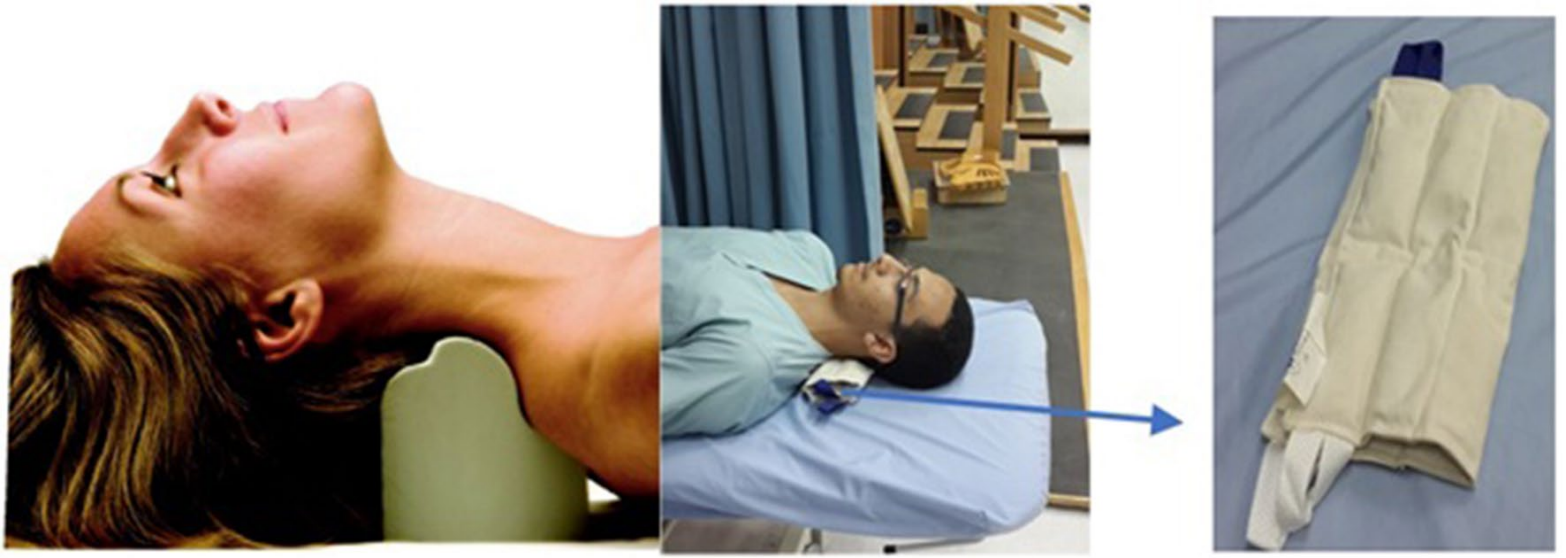
On the far left, the Denneroll orthotic is shown with a mid-cervical spine placement. In the middle, the placebo as a rolled cervical hydrocollator towel is shown placed in the mid-cervical spine. On the far right, a closeup of the placebo treatment is shown for the control group as a cervical hydro-collator towel. Copyright CBP Seminars, Inc. All rights reserved, reprinted with permission, and informed consent from all persons depicted was obtained.

The treating therapist, for both the control and intervention groups, was unblinded to the treatment method but the participants and the examination assessor who conducted the measurements were blinded for their specific group allocation. Further, participants were not allowed to inform the assessor of which treatment they had received, and the assessor was not allowed to ask. For both the placebo and Denneroll intervention, the duration of each session began at three-minutes per session and incrementally increased 1–2 min per session until the goal of 20-min per session was attained. Interventions were applied three times per week for 10-weeks (30 total sessions) in a supervised setting. See Fig. 1 (informed consent to publish the content of this report along with the accompanying images and copyright was obtained from all participants and parties). The entire control or intervention program was delivered individually, face to face by the same physiotherapist, with 20 years of experience who had received training in these techniques; this was done to minimize inter-therapist variation, enhance fidelity and to maintain the same patient physiotherapist relationship. Compliance with the Denneroll and placebo traction interventions was calculated as the total number of sessions attended/total number of sessions available.

### Outcome measures

Multiple outcome measures were obtained at three intervals: pre-treatment, the next day following the completion of the 30 visits, and at 3-months follow-up after the 10-weeks of treatment re-evaluation. The order of measurements was the same for all participants. Radiographic cervical sagittal alignment of AHT and ARA C2–C7 was the primary treatment outcome whereas, neurophysiological variables were the secondary measures.

Standing lateral cervical radiographs were assessed for AHT distance and cervical lordotic angle (ARA C2–C7) via the method investigated by Harrison et al^33,34^.

The secondary neurophysiological findings included central somatosensory conduction time (N13–N20) and amplitudes of the following potentials: spinal N13, brainstem P14, parietal N20 and P27, and frontal N30. For participant neurophysiological assessment, an electromyogram device (Neuropack S1 MEB-9400K, Nihon Koden, Japan) was used to measure these variables. After the skin was cleaned, the stimulating electrodes were placed on the skin overlying the median nerve 2–3 cm superior relative to the distal crease of the wrist. We used a bearable, painless stimulus intensity set at 3 times above the sensory level; no participant reported this as noxious or pain causing^38,39^.

We followed previously published protocols and procedures for the neurophysiological assessments; for recording, careful attention was paid to cleaning and scarifying the skin before the attachment of the recording electrodes to the scalp. Using an impedance below 5 kΩ, the recording electrodes were placed superficial to the sixth cervical vertebra spinous process (Cv6) [anterior neck (AC)]. Additional recording electrodes were placed at the frontal and parietal scalp regions contralateral to the side of stimulation (P3, P4, and F3, F4). The reference electrode was placed ipsilateral to the stimulation site at the earlobe^38,39^.

Each test was repeated at least twice and the summated tracings using two repeatable averages were used for the amplitude and latency measurements. Following previous protocols^38,39^ the band pass was set to 5–1500 Hz, with an analysis time of 100 ms and a band width of 103 μs. A total of 800 sweeps were averaged using electrical square pulse stimuli of 0.2 ms durations. We identified and measured the following potentials:

1. The N13 potential^40^;
2. The far-field P14 potential^40^;
3. The N20 and P27 potentials^41^;
4. The N30 potential^41,42^.

The potential amplitudes were measured from the preceding peak (termed peak-to-peak). There were two different rates of somatosensory evoked potentials (SSEPs), to enable optimal conditions to record the N13, P14, N20, P27 and the N30 SEP peak complexes. The slower rate of 2.47 Hz was optimum for N30 while the faster rate, 4.98 Hz, allowed N13, P14, N20, and P27 to be appropriately quantified.

Using standardized clinical procedures for median nerve stimulation at the wrist, each subject’s central somatosensory conduction time measurement, N13–N20 was determined^43,44^. Differences in peak latencies between N13 and N20 was measured as central conduction time.

### Sample size determination

Estimates of mean and standard deviations were obtained from a pilot project of participants receiving a similar program^35^. The mean difference and 95% confidence interval of the ARA C2–C7 were found to be 13° [− 14.3°, − 11.8°] respectively from this pilot study. Accordingly, at least 36 participants, given a significance level of 5% and statistical power of 80%, were needed in the current study. The calculated sample size was increased by 10% to accommodate for any potential participant drop-out.

### Data analysis

The normal distribution of all descriptive statistics baseline variables was determined using the Kolmogorov–Smirnov test; where continuous data is noted as mean with standard deviation (SD) in the text and tables. Equality of variance was assessed with Levene's test, attaining a 95% confidence level, *P* value > 0.05.

Descriptive statistics (means ± SD unless otherwise stated) are listed at each time point. To identify if group equivalence through randomization was achieved, a Student’s *t *test for continuous variables and chi-squared (for categorical variables) were performed for each demographic and clinical variable.

A 2-way analysis of variance (ANOVA) with repeated measures was utilized to determine the effects of the two unique treatment arms over the course of the investigation at 10-weeks and 3-month follow up. The ANOVA models included an independent variable (group), a repeated measure (time) and an interaction factor (group × time). If interactions were found (*P* < 0.05), paired and independent *t* tests as post hoc analyses were performed. To assess any between group differences, baseline values of all outcome measures as covariates were used.

All results were analyzed with an intention-to-treat approach; with an alpha = 0.05 level of significance. Correlations (Pearson's r) were used to examine the relationships between the amount of change in ARA C2–C7 and AHT and the amount of change in the neurophysiological measures at the three intervals: baseline, 10-weeks, and 3-months follow-up. Additionally, a multiple variable regression analysis to predict central conduction (N13–N20) from ARA C2–C7 and AHT was sought.

Data analysis was performed using SPSS version 20.0 software (SPSS Inc., Chicago, IL, USA) with normality and equal variance assumptions ensured prior to the analysis.

## Results

**Figure 2:**
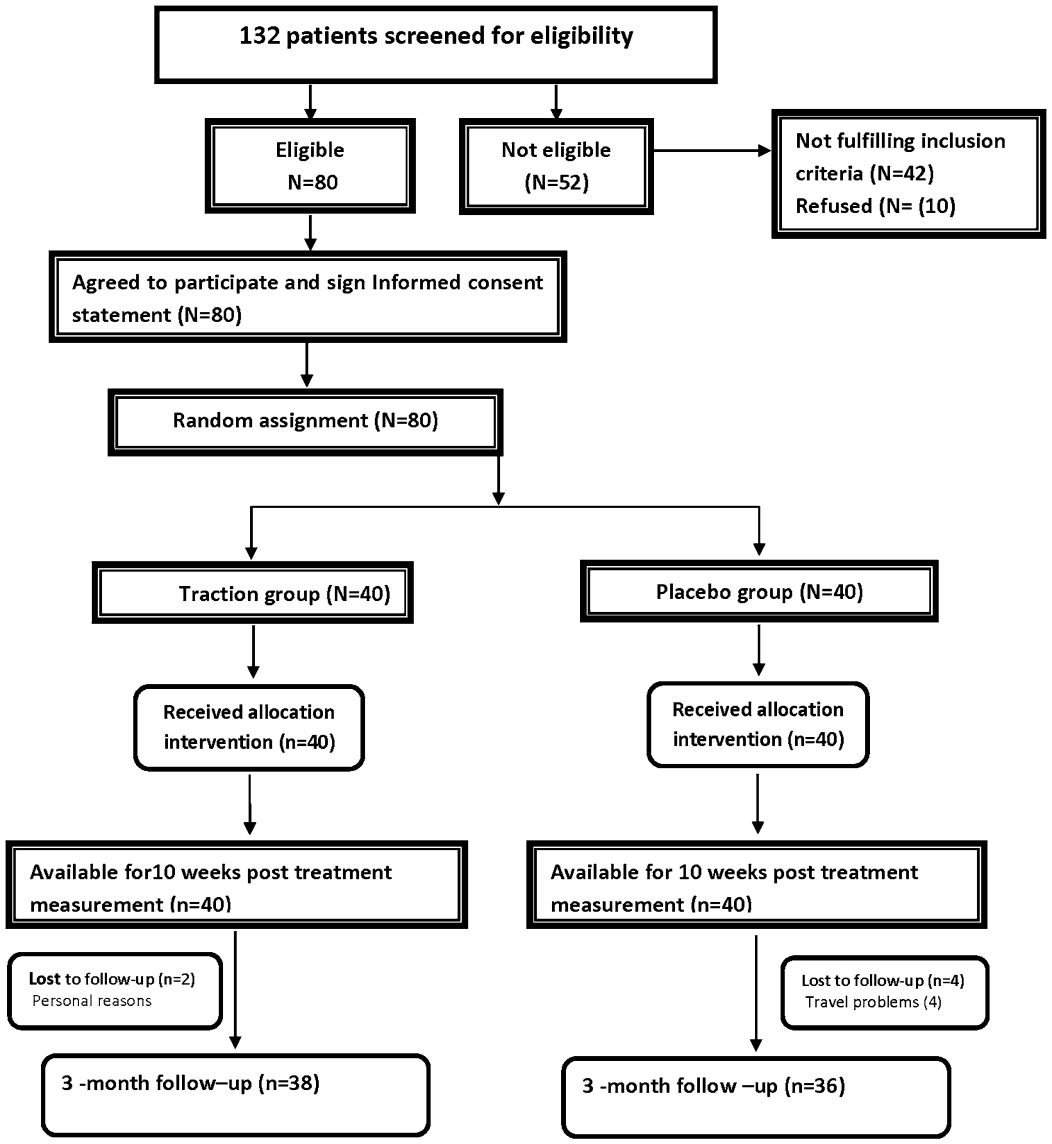
A diagram of patients’ retention and randomization throughout the study.

A flow chart of our study design, participant allocation, and follow through is shown in Fig. 2. A total of 132 potential participants were approached. Of these, 42 (34.4%) did not meet the inclusion criteria, and 10 (7.6%) refused to participate. Eighty persons were eligible to participate in our study. All 80 (100%) participants finished the re-evaluation at 10-weeks/30 sessions of treatment, and 74 participants (92.5%, 38/40 in the intervention and 36/40 in the placebo group) completed the 3-month follow up; indicating 6 participants dropped out after the initial 10-weeks of treatment. All subsequent analysis was by original the assigned groups for participant numbers indicated above.

### Compliance

Compliance with both Denneroll traction and placebo traction interventions was excellent, where participants in the Denneroll traction group attended 93% of sessions. In the placebo traction group, participants attended 90% of sessions. Major reasons for missed sessions were illness (51%) and illness of a family member (49%).

### Sample characteristics

Data for the demographic and clinical variables are presented in Table 1. No statistically significant differences for between group variables were found at baseline, except for N13 (*P* = 0.0022).

**Table 1:** Descriptive data for the demographic and clinical variables are presented.

Variable	Experimental group (n = 40)	Control group (n = 40)	*P *value
Age (years)	21.5 ± 2.7	23.9 ± 3.1	0.07
Weight (kg) pre-treatment treatment	69.2 ± 4.7	72.2 ± 5.1	0.14
**Gender (%)**			
Male	25 (62.5%)	23 (57.5%)	0.4
Female	15 (37.5%)	17 (42.5%)	
**Outcome measures**			
AHT (mm)	2.4 ± 0.4	2.3 ± 0.3	0.5
ARA C2–C7 (°)	4.16 ± 4.15	4.2 ± 4.5	0.7
P14	0.96 ± 0.21	1.045 ± 0.24	0.10
N20	2.44 ± 0.4	2.5 ± 0.38	0.4
P27	1.9 ± 0.27	2.02 ± 0.4	0.10
N30	1.9 ± 0.2	2.1 ± 0.3	0.3
N13–N20	5.7 ± 0.3	5.7 ± 0.3	0.31
N13	1.2 ± 0.16	1.01 ± 0.24	0.002

No statistically significant differences between the control group and the experimental group were found at baseline for their demographic and clinical variables, except for N13 (*P* = 0.0022). The values are presented as mean and standard deviation (SD) for age, weight, anterior head translation (AHT), absolute rotation angle (ARA C2–C7 lordosis), and neurophysiological parameters.

The analysis indicated significant group × time effects at both the 10-week post treatment and the 3-month follow-up in favor of the intervention group on measures of cervical lordosis, anterior head translation, central conduction time (N13–N20) and neurophysiological potential amplitudes of: spinal N13, brainstem P14, parietal N20 and P27, and frontal N30 (*P* < 0.005). The effect sizes (Cohen’s d) were in the medium to large range (Table 2).

**Table 2:** Differences between treatment groups on each outcome measure after 10-weeks of treatment and at 3-month follow up after the 10-weeks of treatment.

Outcome Measure	Group	After 10-weeks of treatment			3-month, 10-week follow-up		
		Mean difference^‡^	(95% CI)	*P*	Mean difference^‡^	(95% CI)	*P*
AHT (Cm)	E vs. C	1.3	[1.09, 1.4]	< 0.005	1.2	[1.086, 1.389]	< 0.005
ARA (°)	E vs. C	− 13.8	[− 15.3, − 12.2]	< 0.005	− 12.7	[− 15.3, − 10.2]	< 0.005
P14	E vs. C	0.34	[0.25, 0.43]	< 0.005	0.31	[0.22, 0.40]	< 0.005
N20	E vs. C	0.46	[0.33, 0.59]	< 0.005	0.51	[0.36, 0.65]	< 0.005
P27	E vs. C	0.5	[0.37, 0.65]	< 0.005	0.6	[0.41, 0.68]	< 0.005
N30	E vs. C	0.49	[0.35, 0.62]	< 0.005	0.45	[0.40, 0.67]	< 0.005
N13	E vs. C	0.2050	[0.11, 0.29]	< 0.005	0.29	[0.21, 0.38]	< 0.005
N13–N20	E vs. C	0.19	[0.06, 0.32]	0.004	0.45	[0.32, 0.57]	< 0.005

Anterior head translation (AHT) in centimeters, lordosis C2–C7 absolute rotation angle (ARA) in degrees, 95% confidence interval (CI).

*E*  experimental group, *C*  control group, *CI* confidence interval.

### Between group analysis at the first follow-up point

The unpaired *t* test analysis revealed significant differences between the intervention and control groups for the previous variables including cervical lordosis, anterior head translation, central condition time (N13–N20), amplitudes of spinal N13, brainstem P14, parietal N20 and P27, and frontal N30 potentials (*P* < 0.0005) (Table 2). These significant differences were maintained at the 3-month follow-up (*P* < 0.005). The effect size (Cohen’s d) for all variables were in the medium to large range (Table 2).

### Within group analysis

After 10-weeks of treatment, the intervention group’s scores identified that differences among column means was significantly greater than expected by chance for all the measured outcomes when compared to baseline values (*P* < 0.0005) (Table 3). The effect size (Cohen’s d) for all variables were in the large range (Table 3).

**Table 3:** Mean and standard deviations for all variables in the experimental and control groups at each evaluation period.

Measure	Group	Baseline mean (SD)	10-Weeks treatment mean (SD)	3-Month follow-up mean (SD)	10-Week versus baseline				3-Month versus baseline			
					Mean difference (95% CI)	*P*	Effect size Cohen *d*	Effect size *r*	Mean diff (95% CI)	*P*	Effect size Cohen *d*	Effect size *r*
AHT (Cm)	E	2.4 ± 0.4	1.01 ± 0.5	1.18 ± 0.5	1.3 [1.153–1.557]	< 0.005	3.06	0.83	1.2 [0.97–1.40]	< 0.005	2.69	0.8
	C	2.3 ± 0.3	2.27 ± 0.2	2.4 ± 0.12	.4 [− 0.02–0.10]	0.19	0.1	0.058	− 0.10 [− 0.16–0.046]	0.21	− 0.43	− 0.21
ARA (°)¤	E	4.16 ± 4.15	18.1 ± 2	17.9 ± 1.9	− 14 [− 14.7–13.24]	< 0.005	− 4.2	− 0.9	− 13.78 [− 14.6–12.9]	< 0.005	− 4.25	− 0.90
	C	4.2 ± 4.5	4.3 ± 4.4	5.1 ± 7.8	− 0.055 [− 0.17–0.059]	0.34	− 0.02	− 0.01	− 0.8925 [− 2.92–1.13]	0.38	− 0.14	− 0.070
P14	E	0.96 ± 0.21	0.68 ± 0.2	0.715 ± 0.18	0.27 [− 0.22–0.32]	< 0.005	1.4	0.57	0.24 [0.21–0.27]	< 0.005	1.36	0.564
	C	1.045 ± 0.24	1.03 ± 0.21	1.03 ± 0.2	0.015 [− 0.008–0.038]	0.2	0.04	0.02	0.015 [− 0.014–0.044]	0.3	0.06	0.033
N20	E	2.44 ± 0.4	2.01 ± 0.26	1.97 ± 0.25	0.43 [0.37–0.49]	< 0.005	1.27	0.53	0.47 [0.415–0.52]	< 0.005	1.40	0.57
	C	2.5 ± 0.38	2.475 ± 0.32	2.5 ± 0.4	0.037 [− 0.0075–0.082]	0.1	0.02	0.013	0.027 [− 0.01–0.066]	0.16	0.0	0.0
N27	E	1.9 ± 0.2	1.4 ± 0.32	1.49 ± 0.29	0.43 [0.32–0.52]	< 0.005	1.96	0.70	0.41 [0.32–0.49]	< 0.005	1.64	0.635
	C	2.02 ± 0.4	2.04 ± 0.3	2.045 ± 0.5	0.025 [− 0.023–0.073]	0.3	− 0.056	− 0.028	− 0.025 [− 0.045–0.004]	0.016	− 0.05	− 0.027
N30	E	1.9 ± 0.2	1.57 ± 0.3	1.57 ± 0.3	0.4 [0.3039–0.5061]	< 0.005	1.29	0.543	0.4 [0.3145–0.4905]	0.007	1.29	0.543
	C	2.1 ± 0.3	2.06 ± 1.06	2.1 ± 0.3	0.03 [− 0.023–0.07]	0.27	0.156	0.07	− 0.027 [− 0.05–0.003]	0.03	0.21	0.107
N13–N20	E	5.7 ± 0.3	5.4 ± 0.4	5.2 ± 0.2	0.282 [0.20–0.36]	< 0.005	0.84	0.39	0.44 [0.41–0.46]	< 0.005	1.90	0.68
	C	5.6 ± 0.27	5.6 ± 0.28	5.7 ± 0.29	0.022 [− 0.003–0.04]	0.08	0.0	0.00	− 0.07 [− 0.13–0.013]	0.03	− 0.35	− 0.17
N13	E	1.2 ± 0.16	0.77 ± 0.09	0.68 ± 0.13	0.39 [0.35–0.43]	< 0.005	0.8	0.37	− 0.4750 [− 0.5418–0.4082]	< 0.005	3.57	0.87
	C	1.01 ± 0.24	0.97 ± 0.25	0.96 ± 0.22	0.035 [− 0.003–0.073]	0.07	0.1	0.08	0.03 [− 0.002–0.06]	0.069	0.21	0.107

Comparison of changes in outcome measures over time within each treatment group.

Anterior head translation (AHT), cervical lordosis absolute rotation angle (ARA) C2–C7.

*E* experimental group, *C* control group, *CI* confidence interval, *SD* standard deviation.

At 10-weeks, the results for the control group showed insignificant changes for all the measured variables AHT (*P* = 0.19), ARA C2–C7 (*P* = 0.34), P14 (*P* = 0.2), N20 (*P* = 0.1), N27 (*P* = 0.3), N30 (*P* = 0.27), N13–N20 (*P* = 0.08), and N13 (*P* = 0.07) (Table 3).

**Figure 3:**
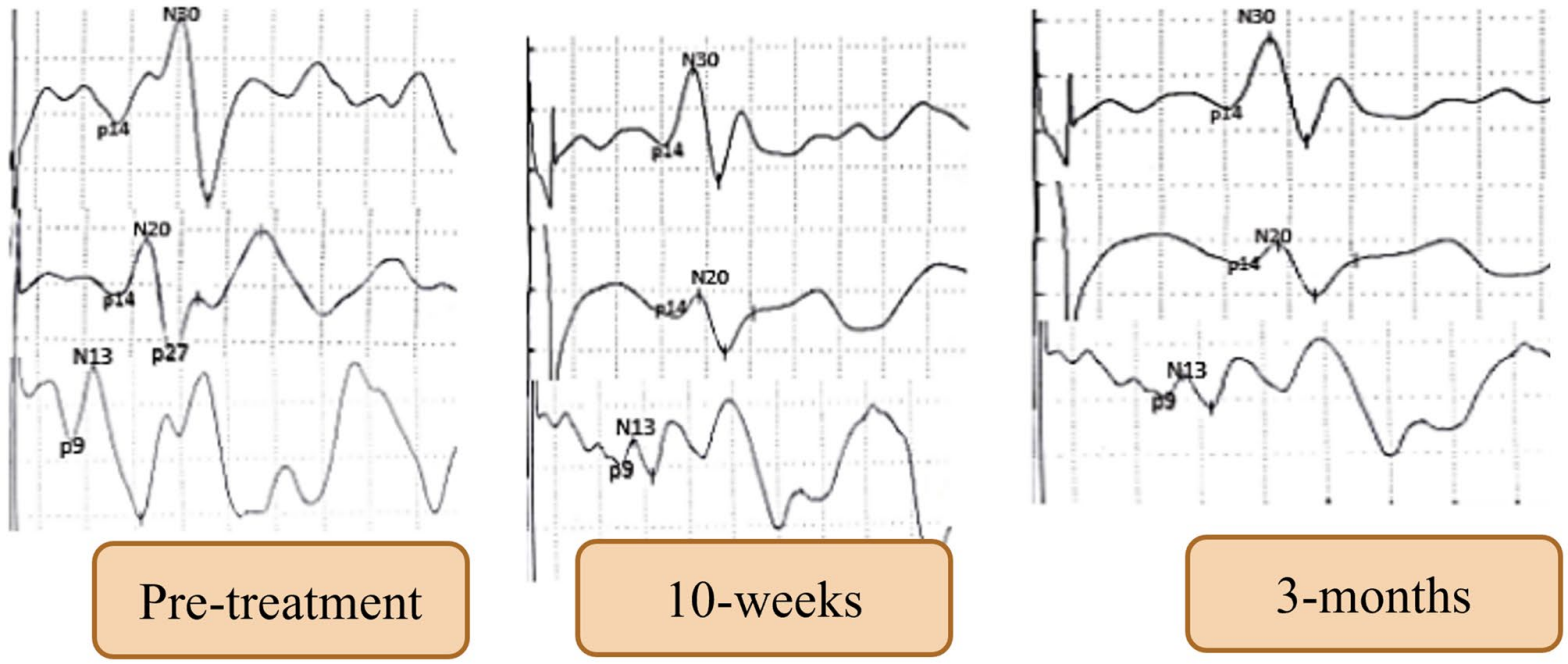
A representative example of spinal, cortical and sub-cortical findings at three intervals of measurement: baseline or pre-treatment, following 10-weeks of treatment, and 3-month follow-up.

**Figure 4:**
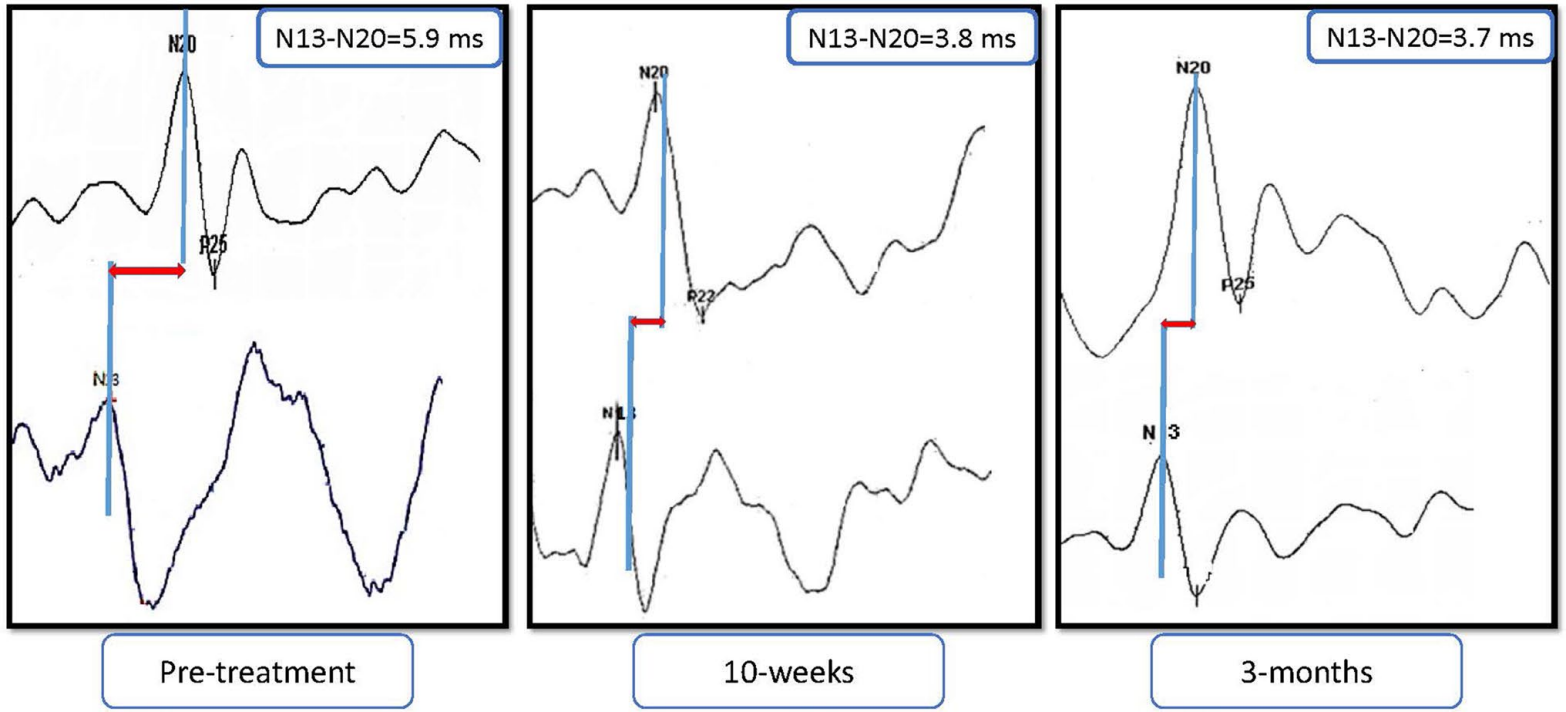
A representative example of central conduction time (N13–N20) at three intervals of measurement: baseline or pre-treatment, following 10-weeks of treatment, and 3-month follow-up. Time is shown in ms.

At 3-month follow-up, analysis of the intervention group’s scores indicated that all the significant improvements from pre-treatment to 10-week post assessment were maintained or extended at the 3-month follow-up interval (*P* < 0.05) (Table 3). The effect size (Cohen’s d) for all variables were in the large range (Table 3). In contrast, the control subject's scores were comparable to their baseline values (Table 3). A representative example of spinal, cortical and sub-cortical findings at the three intervals of measurement for the intervention group is shown in Fig. 3. While Fig. 4 depicts a representative example of central conduction time (N13–N20) at our three examination intervals.

**Figure 5:**
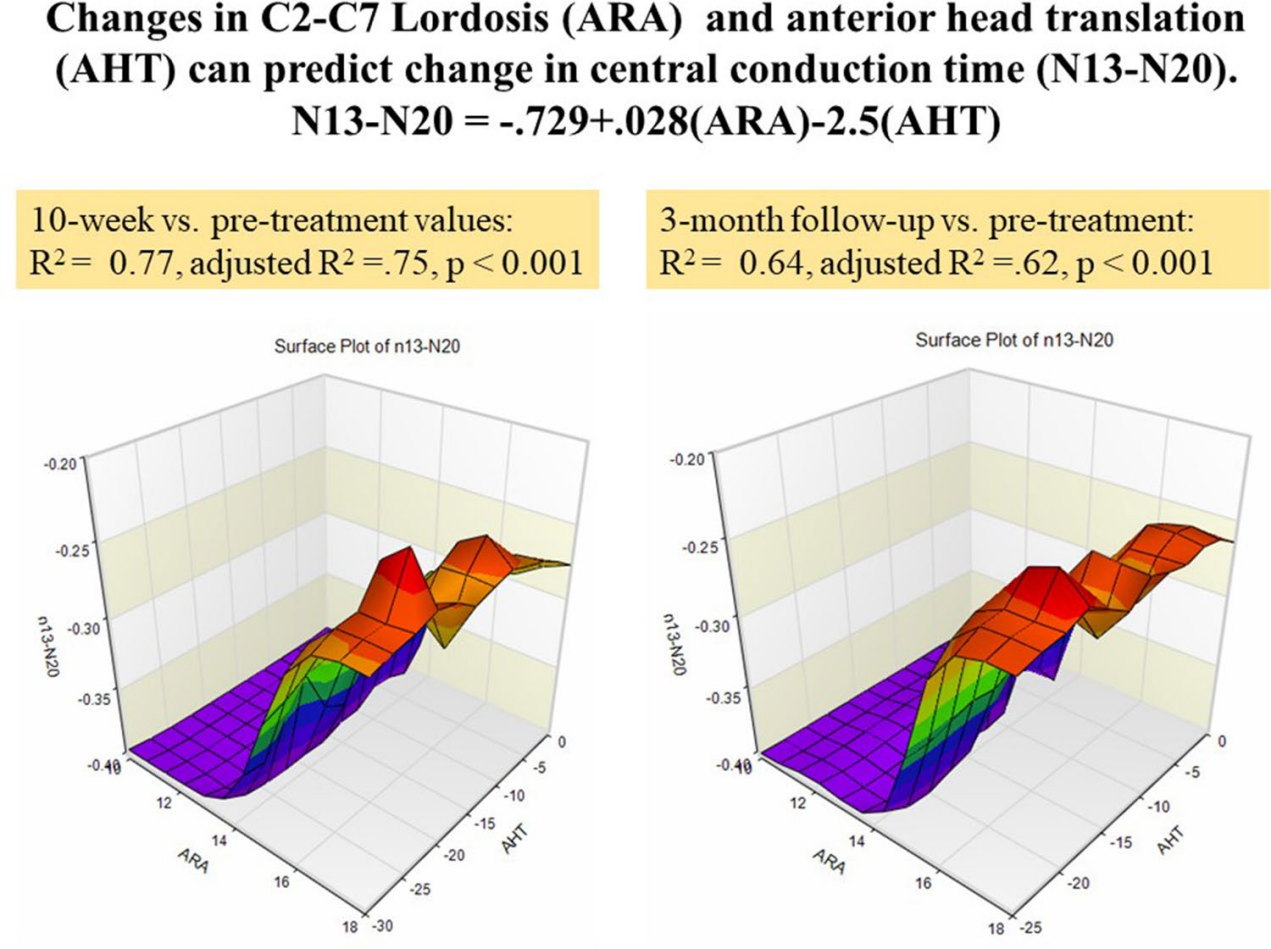
A statistically significant, multiple regression model to predict central conduction N13–N20 from cervical lordosis (absolute rotation angle or ARA C2–C7) and anterior head translation (AHT) was identified for the intervention group as no such relationship existed for the control/placebo group. Left: the 10-week versus pre-treatment (*P* < 0.001). On the right: the 3-month follow-up versus pre-treatment (*P* < 0.001). Note as AHT reduces towards 0 mm and lordosis ARA C2–C7 becomes more lordotic, N13–N20 becomes faster in time.

At 10-weeks, significant negative correlations were identified between the amount of change of the cervical lordotic curve and all the measured variables: amplitudes of spinal N13 (r = − 0.6, *P* < 0.001), brainstem P14 (r = − 0.5, *P* < 0.001), parietal N20 (r = − 0.4, *P* = 0.003), P27 (r = − 0.51, *P* < 0.001), frontal N30 potentials (r = − 0.62, *P* < 0.001), and N13–N20 (r = − 0.47, *P* = 0.003). Also, significant positive correlations were found between AHT distance and the following: N13 (r = 0.63, *P* < 0.001), brainstem P14 (r = 0.53, *P* < 0.001), parietal N20 (r = 0.57, *P* < 0.001), *P*27 (r = 0.67, *P* < 0.001), and frontal N30 potentials (r = 0.59, *P* < 0.001), and N13–N20 (r = 0.6, *P* < 0.001) (Table 4). Lastly, a statistically significant multiple regression model to predict central conduction time changes (N13–N20) from changes in ARA C2–C7 and AHT was identified for the intervention group at both the 10-week mark (*P* < 0.001) and the 3-month follow-up (*P* < 0.001) (Fig. 5).

**Table 4:** Pearson correlation (r) matrix between variables for the intervention group.

	Δ ARA 0–10 W	Δ ARA 10 W–3 Mo	Δ AHT 0–10 W	Δ AHT 10 W–3 Mo
Δ N13–N20 0–10 W	r = 0.47 *P* = 0.003		r = 0.6 *P* < 0.001	
Δ N13–N20 10 W–3 Mo		r = − 0.5 *P* < 0.001		r = 0.54 *P* < 0.001
Δ N13 0–10 W	r = − 0.6 *P* < 0.001		r = 0.63 *P* < 0.001)	
Δ N13 10 W–3 Mo		r = − 0.5 *P* < 0.001		r = 0.7 *P* < 0.001
Δ p14 0–10 W	r = − 0.5 *P* < 0.001		r = 0.53 *P* < 0.001	
Δ p14 10 W–3 Mo		r = − 0.4 *P* = 0.002		r = 0.52 *P* < 0.001
Δ N20 0–10 W	r = − 0.4 *P* = 0.003		r = 0.57 *P* < 0.001	
Δ N20 10 W–3 Mo		r = − 0.6 *P* < 0.001		r = 0.65 *P* < 0.001
Δ p27 0–10 W	r = − 0.51 *P* < 0.001		r = 0.67 *P* < 0.001	
Δ p27 10 W–3 Mo		r = − 0.63 *P* < 0.001		r = 0.5 *P* < 0.001
Δ N30 0–10 W	r = 0.62 *P* < 0.001		r = 0.59 *P* < 0.001	
Δ N30 10 W–3 Mo		r = 0.56 *P* < 0.001		r = 0.67 *P* < 0.001

Anterior head translation (AHT), cervical lordosis absolute rotation angle (ARA) C2–C7, Δ = change in the variable over time shown.

**Δ 0**–**10 W:** the amount of change between initial base line and 10-weeks of treatment.

**Δ 10 W**–**3 Mo:** the amount of change between the 10-weeks of treatment values and 3-month follow-up.

No adverse reactions or events were reported by any of the participants in either group during the time frame of this trial.

## Discussion

The differences between our intervention and control groups central conduction time and other neurophysiological potential amplitudes, indicates that cervical lordosis and sagittal posture improvement did alter central conduction time and central processing in an asymptomatic population. Thus, our study's primary hypothesis is confirmed by these findings. To our knowledge this is the first study to provide objective evidence that the alignment of the sagittal cervical curve influences these specific neurophysiological measures in asymptomatic participants.

### Cervical lordosis improvements

In the intervention group receiving the Denneroll traction, the cervical lordosis increased (mean of 13.1° after 30 treatment sessions) and was maintained at 3-months. In contrast, we identified no significant improvement in the control group’s cervical lordosis at any of the follow-up evaluations compared to baseline assessment after 30 sessions on a placebo rolled towel. Our intervention group's results agree with the findings reported by Moustafa et al^20,35–37^. It is likely that orthotics such as the Denneroll (and other 3-point-bending traction devices) cause visco-elastic creep deformation of the anterior soft tissues (musculature, longitudinal ligament and intervertebral discs), resulting in ‘unloading’ of the disc and normalized load sharing on the cervical column^20,35–37^.

### Cortical, subcortical and spinal neural changes

We identified neuroplastic changes, likely resulting from alterations in cervical sagittal alignment, that occur at various levels of the somatosensory system. These findings are supported by other studies which reported that subcortical changes are important drivers of cortical reorganization^45,46^.

Specific to symptomatic patient cases, spinal, cortical, and subcortical reorganizational processes in humans have been confirmed in cases with abnormal afferentation processes^38,39^. Generally, our study findings are supported by earlier investigations describing that the reorganization of the somatosensory system is primarily driven by alterations or modifications of sensory input^29–32^.

The assumption that restoring normal posture and cervical spine alignment is important for a better afferentation process has some preliminary evidence. For instance, modeling studies have predicted that as AHT increases, increased stress and strain is placed upon the muscles and ligaments of the cervical and thoracic region^47^. This abnormal head posture results in altered joint position and dysfunction that may lead to abnormal neurophysiologic afferent information (so called dysafferentation)^19,20^. Furthermore, altered cervical lordosis and increased anterior head translation can result in increased physical demands causing premature and acceleration of degenerative changes in the muscles, ligaments, bone and neural tissues^47–50^.

While there is considerable controversy over the significance of degenerative spine changes and their correlation to patient pain and disability^51,52^, a recent meta-analysis, detailing 14 studies covering 1193 asymptomatic persons matched to 1904 symptomatic persons (up to 50 years of age) identified that disc degeneration, extrusion, protrusion, modic-1 changes, and vertebral body spondylolysis are all more prevalent in adults with back pain compared with asymptomatic individuals^52^. Regardless of the exact correlation between spine degeneration and the presence of pain, there is preliminary evidence that spine degeneration can compromise the neuromuscular stabilizing system of the spine^53^. Additionally, disc degeneration, altered cervical lordosis, and forward head translation can all contribute to a reduced range of movement and an altered segmental cervical spine kinematic pattern^53–55^. Therefore, it is likely that altered sagittal cervical spine alignment, in the form of FHP and abnormal cervical curvature, are drivers of altered sensorimotor integration through an altered afferent input from altered cervical spine kinematics and altered soft tissue strains; this explains some of the results from both case control studies^19^ and randomized trials on the topic^20^.

### Central Somatosensory conduction time

Mechanically, increased longitudinal strain acting on the spinal cord tissues might be a primary mechanism leading to the neurologic dysfunction as identified in our investigation. Specifically, spinal cord biomechanics might explain our finding of more efficient central conduction time in our intervention group with increased lordosis post intervention^49,50^. For example, neural axoplasm has thixotropic properties, indicating that a lack of motion and/or increased constant strain might increase the viscosity and potentially slow or impair neural transport mechanisms^56^.

Furthermore, neural tissue needs ample amounts of oxygen and various other nutrients to maintain optimum structure and function^57^. There is evidence to suggest that abnormal sagittal cervical alignment may place a constant, increased strain on neural and vascular elements in the cervical spine, directly impairing the blood flow to the spinal cord, nerve roots and cerebral areas^58–60^. For example, Katz et al^61^ identified a linear correlation between cervical lordotic curve magnitude induced on the cervical denneroll orthtoic and increased MRI-angiogram cerebral blood flow pixel intensity.

Similarly, in 1988, Tasaki^62^ proposed a new mechanical and chemical model of axonal action potentials. Although involved, two primary mechanical changes were identified in the excitation process: first, there is a rise in the internal presure of the axon causing a three-dimensional cross-sectional swelling and second, a longitudinal shortening of the axon is caused by the internal pressure rise. More recently, researchers have identified that neural impulses function similarly to a sound pulse or “soliton”; as the signal propagates along the pathway, the membrane changes to a more solid form that, due to slight compression, generates a piezoelectric pulse^63,64^. Thus, the electrical event is not only based on biochemical interactions, it is caused by mechanical forces as well. It is theoretically possible, therefore, that increased longitudinal stress and strain on the spinal cord due to a straightened or reversed cervical curve may subtly impair the mechanical excitation events that are known contributions to the production and propogation of neural action potentionals.

Relatively few studies exist, however, supporting this mechanical concept; though one study reported a significant reduction of afferent and efferent impulse conduction, detected by somatosensory-evoked potentials and neurogenic motor-evoked potentials after spinal cord distraction^59^. Other studies suggest that cervical kyphosis or flexion may indeed result in various neuronal dysfunctions either from longitudinal or cross-sectional (shear) stress and strains^16–18,49,50^.

## Study limitations

We propose several limitations of this study pointing to future works. First, our project only included a short-term follow-up of 3-months after intervention was terminated; it is unclear how long the identified neurophysiological changes would remain. Second, our choice of the hydrocollator towel as the placebo might have influenced our results in the control group. However, the rolled hydrocollator towel’s ridges or bumps cause contact pressure on the posterior aspect of the patient’s cervical spine and this was chosen to mimic the ridge on the Denneroll device but without creating significant extension bending. It is unknown if a different placebo would have different results in the control group.

Additionally, at the start of the investigation the N13 potential, recorded at Cv6 originating in the dorsal horn of the cervical spinal cord (21), showed statistically significant differences between our two groups; however, N13–N20 conduction time was not different between the groups, so we do not believe this impacted the significance of our study's findings. Fourth, because we chose a young adult, asymptomatic/healthy population to investigate, it is unknown how our study results might relate to musculo-skeletal pain populations. However, while it is standard of care for the rehabilitation of patients with spinal disorders to be based on improving functional impairments, limitation of activities and restriction of social activities^65^, it would seem appropriate to integrate structural rehabilitation (improvement in sagittal posture and curvature) in the treatment of spinal disorders. In fact, several recent randomized trials have identified that patients improve to a greater extent at long-term follow-up when their cervical lordosis and FHP are improved^5,20,35–37^. Similarly, if central conduction time and processing can be improved by adding sagittal plane spine correction to the clinical armamentarium of treatment methodology, then this would seem prudent. Previously Moustafa and colleagues^35^, investigated sagittal plane posture and curve correction added to a multi-modal program for patients with chronic pain and impairments resulting from cervical disc herniation. They^35^ identified a more efficient, approximate 20% faster, N13–N20 central conduction time in the group receiving cervical sagittal plane correction; the results obtained in the current study confirm the validity of this^35^, preliminary finding.

Fifth, it was noted that some participants in the intervention group had increased skull extension on their re-evaluation lateral cervical spine radiographs; the value of this increased extension was always less than 14° and often normalized to horizontal on the 3-month follow-up. The increased skull extension on post-treatment radiographs in the intervention group is due to the extension bending effect of the cervical Denneroll. Although some authors opine that this skull extension creates reliability and validity concerns for interpreting post treatment improvements in cervical lordosis, this is likely not the case kinematically^66^. Also, the result of our multiple regression modelling indicates that, regardless of why the participants in the intervention group have increased lordosis and improved posture, central conduction time is more efficient with improved cervical sagittal posture.

Finally, our investigation did not include a natural history group such that we were unable to detect and measure the placebo effect vs. the natural history^67^. However, at the time of this investigation, we considered it too difficult to truly blind the patients to the protocols in this investigation. Herein, the investigator could not be blinded to the group allocation of receiving the Denneroll orthotic vs. the placebo versus natural history.

## Future studies

As our investigation is the first of its kind addressing the effects of sagittal curve correction on central conduction time and central processing in an asymptomatic population there are several areas of future investigations needed to address the limitations presented above. For example, participant prior experiences and expectation perceptions need to be explored. Additionally, the credibility of the hydrocollator placebo needs to be investigated against a true natural history control population. Similarly, the main contextual factors with the potential to influence and affect the different neurophysiological outcomes should be explored. Investigations need to explore the influence of different pain syndromes on the neurophysiological outcomes reported herein to determine which specific population might benefit the most or the least from sagittal cervical alignment rehabilitation. Lastly, it remains to be determined how improved central conduction times, as measured with somatosensory evoked potentials, translates into improved patient outcomes across a variety of measurements (pain, disability, sensorimotor control, health status, etc.).

## Conclusion

We identified a reduced cervical lordosis and anterior head translation is associated with differences in neural activity at several regions (cortical and subcortical) of the somatosensory system. Restoration of the cervical sagittal alignment, in terms of cervical lordosis and anterior head translation, has a direct influence on the central conduction time. Clinical interventions directed at improving central processing through restoring the normal sagittal alignment could be added to clinical interventions targeting specific spinal disorders.

## Data Availability

The datasets generated and/or analyzed during the current study and the full trial protocol are available from the lead author’s University department on reasonable request.
